# Radiation dosimetry of para-chloro-2-[^18^F]fluoroethyl-etomidate: a PET tracer for adrenocortical imaging

**DOI:** 10.1186/s13550-024-01109-2

**Published:** 2024-05-21

**Authors:** Isabella Silins, Adrian Moreno, Anders Wall, Franklin Aigbirhio, Mark Gurnell, Morris Brown, Sara Roslin, Gunnar Antoni, Per Hellman, Anders Sundin, Mark Lubberink

**Affiliations:** 1 Department of Surgical Sciences, Uppsala University, Uppsala, SE-751 85 Sweden; 2 Department of Medicinal Chemistry, Uppsala University, Uppsala, Sweden; 3 Wolfson Brain Imaging Centre, University of Cambridge, Cambridge, UK; 4 Institute of Metabolic Science & Department of Medicine, University of Cambridge, Cambridge, UK; 5 William Harvey Heart Centre, Queen Mary University of London, London, UK

**Keywords:** [^18^F]CETO, Positron emission tomography, Primary aldosteronism, Adrenal, Tumour, Adenoma, Conn adenoma

## Abstract

**Background:**

[^11^C]metomidate, a methyl ester analogue of etomidate, is used for positron emission tomography of adrenocortical cancer, and has been tested in recent clinical trials for lateralization in primary aldosteronism (PA). However, in PA, visualization as well as uptake quantification are hampered by the tracer’s rather high non-specific liver uptake, and its overall clinical usefulness is also limited by the short 20-minute half-life of carbon-11. Therefore, we evaluated para-chloro-2-[^18^F]fluoroethyl-etomidate, [^18^F]CETO, a fluorine-18 (T_1/2_=109.8 min) analogue, as a potential new adrenocortical PET tracer. The aim of this study was to assess radiation dosimetry of [^18^F]CETO.

**Results:**

[^18^F]CETO showed a high uptake in adrenal glands, still increasing at 5 h post injection. Adrenal glands (absorbed dose coefficients 0.100 ± 0.032 mGy/MBq in males and 0.124 ± 0.013 mGy/MBq in females) received the highest absorbed dose. The effective dose coefficient was 20 µSv/MBq.

**Conclusions:**

[^18^F]CETO has a favourable biodistribution in humans for adrenal imaging. The effective dose for a typical clinical PET examination with 200 MBq [^18^F]CETO is 4 mSv.

**Trial registration:**

ClinicalTrials.gov, NCT05361083 Retrospectively registered 29 April 2022. at, *URL*: https://clinicaltrials.gov/ct2/show/NCT05361083.

## Introduction

Adrenocortical pathologies comprise a range of conditions such as benign adrenocortical adenomas, adrenal hyperplasia and adrenocortical carcinomas [1]. The positron emission tomography (PET) tracer [^11^C]metomidate ([^11^C]MTO) shows high specific binding to enzymes encoded by the genes CYP11B1 (11ß-hydroxylase) and CYP11B2 (aldosterone synthase) [2, 3]. [^11^C]MTO PET has been proposed as an alternative to adrenal vein sampling for the lateralization of primary aldosteronism (PA) [4]. The hallmark of PA is an increased secretion of aldosterone by the adrenal cortex, caused primarily by either an aldosterone-producing adrenocortical adenoma or by bilateral adrenal hyperplasia. Elevated aldosterone levels cause the synchronous decrease of renin, resulting not only in hypervolemic hypertension but also in an increased aldosterone-to-renin ratio. Consequently, aldosterone-to-renin ratio is used as the screening method of choice for PA [5]. Many variables may interfere with the results, reducing the sensitivity of the method. Therefore, additional confirmatory testing is required [6]. However, all the confirmatory methods in current clinical use also suffer from their own respective limitations, ultimately prompting only the most apparent cases to receive a PA diagnosis [7, 8]. Globally, PA causes between 3% and 13% of all hypertension cases: an accurate as well as readily available diagnostic technique is essential [9, 10].

PET imaging may provide a valuable diagnostic tool, but in the clinical setting [^11^C]MTO is sparsely used. The clinical availability of [^11^C]MTO is restricted to university hospitals with their own in-house cyclotron due to the short half-life of carbon-11 (T_1/2_ = 20.4 min) [11]. Its usefulness is further hampered by significant tracer accumulation in the liver, which, due to its proximity to the right adrenal gland, may obfuscate adrenal pathology. Additionally, occasional tracer uptake in various liver lesions [12] may provide false-positive imaging results. For [^11^C]MTO PET to be of use in the lateralization of PA, a few days of dexamethasone pre-treatment is generally necessary, as [^11^C]MTO binds to both 11ß-hydoxylase and aldosterone synthase [4]. Hence, there is a need to develop a new PET tracer, preferably exhibiting both extended half-life and lower liver uptake.

We have developed para-chloro-2-[^18^F]fluoroethyl-etomidate ([^18^F]CETO), which has relevant characteristics superior to those of [^11^C]MTO. [^18^F]CETO binds to the adrenal cortex with high specificity and exhibits more favourable in-vivo binding properties than [^11^C]MTO [13]. Also, its non-specific signal in the liver is lower than that of [^11^C]MTO, allowing for better visualization of the right adrenal gland as shown in a recently published first-in-human study evaluating the kinetics of [^18^F]CETO in healthy adrenals and adrenocortical tumours [14]. Furthermore, the longer half-life of [^18^F]CETO has a clear advantage over [^11^C]MTO, simplifying logistics. The aim of the present work was to estimate the biodistribution and radiation dosimetry of [^18^F]CETO in humans.

## Materials and methods

### Subjects

Nine patients with a range of adrenal pathologies (primary aldosteronism, cortisol-producing adenoma, non-functioning adrenal adenoma) were included in this study (mean age 54, range 44–68 years; four females & five males). Patient characteristics are given in Table 1.

**Table 1:** Subject demographics

Patient number	Sex	Age	Weight (kg)	Length (cm)	Diagnosis (L/*R* adrenal)
1	M	68	86	170	Normal/PA
2	F	64	90	162	NFA/NFA
3	M	59	93	188	Cushing/Normal
4	F	44	115	170	Normal/PA
5	M	51	87	172	PA/Normal
6	F	50	85	171	Cushing/Normal
7	F	51	94	171	Normal/PA
8	M	55	99	168	Normal/PA
9	M	47	81	183	Myelolipoma/Normal

PA: primary aldosteronism, NFA: non-functioning adenoma

### PET protocol

[^18^F]CETO was produced according to previously described methods [13]. A 90-minute list-mode PET acquisition was started over the upper abdomen simultaneously with the injection of 2.7 ± 0.4 MBq/kg body weight of [^18^F]CETO (mean 246 MBq, range 205–337 MBq). Thereafter, three whole-body PET/CT examinations ranging from the base of the skull to the proximal thighs were performed at approximately 2 h (2 min/bed position), 3 h (3 min/bed position) and 5 h (5 min/bed position) post injection (p.i.). All examinations were performed on a 4-ring Discovery ST PET/CT scanner (GE Healthcare, Waukesha, Wisconsin, USA) [15]. Attenuation correction was based on an ultra-low-dose CT, and the PET images were reconstructed using time-of-flight ordered subset expectation maximization including resolution recovery using 3 iterations, 16 subsets, and a 3 mm Gaussian post-filter, applying all appropriate corrections to ensure quantitatively accurate images. The dynamic scan was reconstructed into 37 frames of increasing duration (1 × 10, 8 × 5, 4 × 10, 2 × 15, 3 × 20, 4 × 30, 5 × 60, 4 × 300, 6 × 600 s). Patients were requested to void their bladder between examinations, and the total urine volume at each voiding and the radioactivity concentration in the urine were measured. Cross-calibration of the PET scanner with the absolutely calibrated dose calibrator used to measure injected activity, as well as the well-counters used to measure urine samples, were verified on a monthly basis and a new calibration was performed every three months or whenever a calibration error larger than 3% was found.

### Volumes of interest

Volumes of interest (VOIs) were drawn separately over the dynamic scan and each of the whole-body images using VOIager 4.0.7 software (GE Healthcare, Uppsala, Sweden). VOIs over the whole-body scans were not identical to VOIs over the dynamic images as subjects left the scanner between scans, but they were drawn in a consistent manner across scans. In the dynamic scans, VOIs were drawn over representative parts of organs in those frames where each organ (adrenal glands, stomach, blood, kidneys, liver, pancreas, vertebral body, spleen, gallbladder and urinary bladder) was best visualized and then projected over all frames to create time-activity curves (TACs), which were then completed with the values of the three whole-body scans. Standardized uptake value (SUV) curves were calculated by normalization to injected activity per body weight.

### Time-integrated activity coefficient (TIAC) calculations

All TACs were uncorrected for decay. The area under the curve (AUC) of the dynamic imaging portion of each TAC was calculated by rectangular integration, i.e. multiplication of the value in each frame of the dynamic scan with the corresponding frame duration. A single exponential fit to the last data point in the dynamic scan and the three whole-body scans was used to estimate the remainder of the AUC. The only exception to this was the gallbladder contents, for which an exponential fit to data from the three whole-body scans was combined with trapezoidal integration between the end of the dynamic scan and the first whole-body scan. The time-integrated activity was calculated as the AUC under each TAC and multiplied by its respective organ weight by proportional scaling of the adult male or female reference organ weights in OLINDA/EXM 1.1 [16] based on each patient’s body weight relative to the reference phantom body weights. Vertebrae uptake was attributed completely to red marrow. TIACs were calculated by division with the amount of injected activity. The gastrointestinal tract model of the International Commission on Radiological Protection (ICRP 30) [17] was used to obtain TIACs for stomach wall, small intestine (SI), upper (ULI) and lower large intestine (LLI) based on the fraction of injected activity entering the stomach. Urinary bladder TIAC was calculated based on the measured urine volumes and radioactivity concentrations, assuming linear bladder filling between voidings and a voiding interval of 4 h after the last measurement as previously described [18]. Remainder of the body TIAC was calculated as the theoretical maximum residence time minus all source organ residence times and the activity cleared though bladder voiding.

### Dosimetry calculations

Absorbed dose calculations were made using OLINDA/EXM 1.1 [16]. Effective doses were calculated according both to the weight factors in ICRP 60, as available in OLINDA/EXM 1.1, and by applying the ICRP 103 weight factors [19] to the absorbed organ doses calculated by OLINDA/EXM 1.1.

## Results

Table 1 shows demographics and clinical information of the included subjects. No side effects or discomforts were reported by the patients after the [^18^F]CETO injection or during the 5-hour imaging study.

**Fig. 1:**
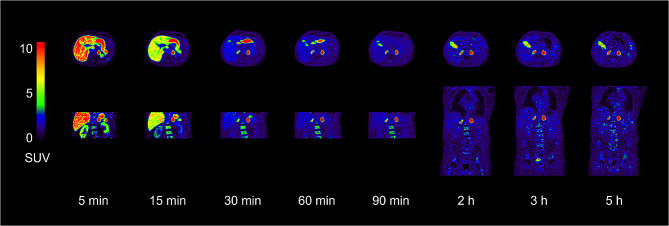
Representative transaxial (upper panel) and corresponding coronal images (lower panel) in a human subject at 5, 15, 30, 60, 90, 120, 180 and 300 min after [^18^F]CETO administration. The tracer is almost exclusively accumulated in the adrenal cortex and is excreted through the kidneys

**Fig. 2:**
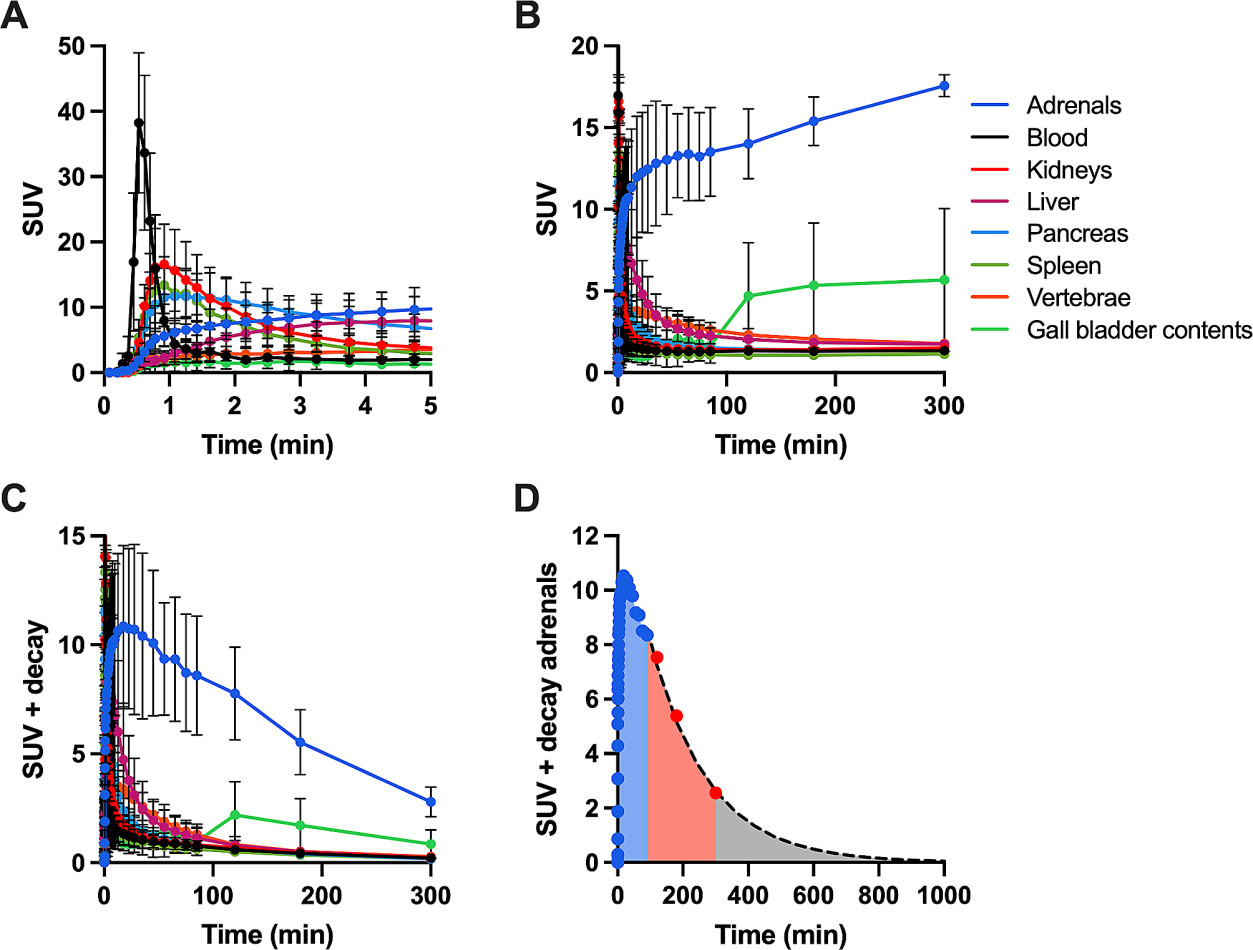
[18 F]CETO SUV versus time time in all source organs with (**A**, **B**) and without decay correction (**C**). Error bars denote inter-subject standard deviations. Estimation of the area under the curve (AUC) in the adrenals is illustrated in (**D**). The blue shaded area depicts the exact AUC under the TAC of the dynamic scan; the dashed line the exponential fit to the four last data points; the red shaded area the AUC under the exponential fit until the last measurement and the grey shaded area the extrapolated part of the AUC

Figure 1 shows coronal and transaxial [^18^F]CETO PET/CT images at 5, 15, 30, 60, 120, 180 and 300 min after injection, primarily showing a high uptake in the adrenal glands. Physiological accumulation is also seen in the stomach at 15, 30 and 60 min, as well as in the gallbladder at later time points. Figure 2 shows SUV versus time curves, with and without decay correction, in all source organs. A rapid clearance after initial uptake followed by near constant SUV values after the first 90 min was seen in all organs except adrenals and gallbladder contents, in which [^18^F]CETO uptake was still increasing by the end of the PET/CT examinations.

Effective half-lifes after 90 min ranged from 81 ± 23 min in vertebrae to 140 ± 18 min in adrenals (Table 2). Figure 2D shows an example of the estimation of the AUC used to estimate the TIACs. Table 3 shows the TIACs. Complete urinary data were only available for five subjects, so the mean TIAC of these five subjects was applied in the effective dose calculations for the remaining four subjects. Estimates of absorbed organ dose coefficients are given in Table 4. The highest aborbed dose was seen in the adrenals, followed by gallbladder wall, liver, and urinary bladder wall. The effective dose coefficient for [^18^F]CETO in humans was 0.020 mSv/MBq using ICRP 103 organ weight factors, corresponding to 4 mSv effective dose for a typical administration of about 200 MBq as used in the present study.

**Table 2:** Extrapolated fraction of TIACs and effective half-lifes

Organ	Extrapolated fraction (%)	Half-life (min)
Adrenals	24 ± 4	140 ± 18
Blood	15 ± 6	118 ± 24
Kidney	15 ± 3	119 ± 17
Liver	7 ± 3	88 ± 17
Pancreas	8 ± 2	94 ± 12
Vertebrae	7 ± 4	81 ± 23
Spleen	14 ± 4	119 ± 21
Gallbladder contents	30 ± 18	116 ± 30
Urinary bladder contents	39 ± 16	n.a.

**Table 3:** Time-integrated activity coefficients

Organ	TIAC male (*N* = 5) (mean ± SD, h)	TIAC female (*N* = 4) (mean ± SD, h)	TIAC all (mean ± SD, h)
Adrenals	0.010 ± 0.003	0.010 ± 0.001	0.010 ± 0.002
Gallbladder contents	0.011 ± 0.006	0.007 ± 0.004	0.009 ± 0.005
Heart contents	0.024 ± 0.007	0.027 ± 0.004	0.025 ± 0.006
Kidneys	0.019 ± 0.005	0.023 ± 0.004	0.020 ± 0.005
Liver	0.172 ± 0.080	0.144 ± 0.040	0.160 ± 0.063
Pancreas	0.006 ± 0.002	0.006 ± 0.001	0.006 ± 0.001
Spleen	0.009 ± 0.003	0.009 ± 0.002	0.009 ± 0.002
Red marrow	0.084 ± 0.032	0.133 ± 0.027	0.106 ± 0.038
Stomach contents	0.019 ± 0.005	0.019 ± 0.004	0.019 ± 0.004
SI contents	0.030 ± 0.008	0.031 ± 0.006	0.030 ± 0.007
ULI contents	0.018 ± 0.005	0.017 ± 0.003	0.017 ± 0.004
LLI contents	0.003 ± 0.001	0.003 ± 0.001	0.003 ± 0.001
Urinary bladder contents	0.025 ± 0.002	0.025 ± 0.005	0.025 ± 0.003
Remainder of the body	2.19 ± 0.11	2.17 ± 0.05	2.18 ± 0.08

**Table 4:** Absorbed dose and effective dose coefficients

Organ	Male (*N* = 5) mean ± SD (mGy/MBq)	Female (*N* = 4) mean ± SD (mGy/MBq)
Adrenals	0.100 ± 0.032	0.124 ± 0.013
Brain	0.010 ± 0.000	0.012 ± 0.000
Breasts	0.009 ± 0.000	0.012 ± 0.000
Gallbladder wall	0.033 ± 0.008	0.031 ± 0.007
LLI wall	0.015 ± 0.001	0.018 ± 0.001
SI	0.019 ± 0.002	0.023 ± 0.002
Stomach wall	0.020 ± 0.002	0.024 ± 0.002
ULI wall	0.021 ± 0.002	0.024 ± 0.001
Heart wall	0.018 ± 0.002	0.022 ± 0.001
Kidneys	0.019 ± 0.003	0.024 ± 0.003
Liver	0.026 ± 0.010	0.030 ± 0.006
Lungs	0.012 ± 0.000	0.015 ± 0.000
Muscle	0.011 ± 0.000	0.014 ± 0.000
Ovaries	-	0.018 ± 0.000
Pancreas	0.022 ± 0.003	0.025 ± 0.002
Red marrow	0.017 ± 0.002	0.022 ± 0.002
Osteogenic cells	0.020 ± 0.001	0.029 ± 0.001
Skin	0.009 ± 0.000	0.011 ± 0.000
Spleen	0.016 ± 0.003	0.020 ± 0.002
Testes	0.011 ± 0.000	-
Thymus	0.012 ± 0.000	0.015 ± 0.000
Thyroid	0.011 ± 0.000	0.013 ± 0.000
Urinary bladder wall	0.024 ± 0.001	0.029 ± 0.003
Uterus	-	0.018 ± 0.000
Total body	0.012 ± 0.000	0.015 ± 0.000
Effective dose (mSv/MBq) ICRP 60	0.017 ± 0.001	0.021 ± 0.000
Effective dose (mSv/MBq) ICRP 103	0.018 ± 0.001	0.022 ± 0.000

## Discussion

[^18^F]CETO is a new and promising ^18^F-labelled adrenocortical targeting PET tracer [13, 14]. In this study, we present human biodistribution and dosimetry results of [^18^F]CETO in nine subjecys with functioning and non-functioning adrenocortical adenomas.

The effective dose for a typical clinical [^18^F]CETO scan using 200 MBq would amount to 4 mSv. The main clinically used alternative to [^18^F]CETO is [^11^C]MTO, but to the best of our knowledge no dosimetry results for [^11^C]MTO have been published. Due to the shorter half-life of ^11^C, the effective dose per MBq is likely lower for [^11^C]MTO. For comparison, an effective dose of 1.9–3.2 mSv per administration has been reported for the single-photon emission computed tomography (SPECT) tracer [^123^I]IMTO [20].

The main sources of uncertainties in the TIACs are the extrapolation of the measured data beyond the last measurement point and the scaling of activity concentration in representative parts of organs to whole organ activity. The mean fraction of the TIACs due to extrapolation beyond the last measurement point ranged from 7% for liver to 30% for gallbladder contents and 39% for urinary bladder contents (Table 2). Mean effective half-lifes after 2 h p.i. were higher than the physical half-life of ^18^F in adrenals, blood, kidneys and gallbladder contents. In adrenals, kidneys and gallbladder this is due to continuing accumulation of [^18^F]CETO at the last measurement point. The effective half-life in blood, slightly above the physical half-life, may be due to measurement uncertainty but could also be due to the release of radioactive metabolites from the liver. In general, as illustrated in Fig. 2D, the kinetics during the final four measurements were well approximated by a single exponential fit, except for gallbladder contents. As the dynamic scan directly measures the AUC during the first 90 min, and only a limited fraction of the AUC was based on extrapolation, the uncertainty in AUC values is likely much smaller than the inter-subject variation in TIACs shown in Table 3.

The estimation of total organ activity based on scaling of reference phantom organ masses by body weight in relation to the reference phantoms is an additional source of uncertainty. Definition of VOIs comprising whole organs, as recommended in the recent EANM guidance on dosimetry for first-in-human studies [21], would avoid the need for scaling to obtain total organ activities. However, whole-organ VOIs suffer from partial volume effects, are often not feasible because entire organs are not within the field of view during dynamic scans, and, when drawn on CT images, may be inaccurate due to for example respiratory motion-related PET/CT misalignment. In general, organ sizes scale with body height rather than weight, and most included subjects had a relatively large body mass index which would lead to an overestimation of organ weights in our results. As scaling with height would have resulted in lower TIACs, the presented absorbed dose and effective dose coefficients may slightly overestimate the true values.

Absorbed dose coefficients for salivary glands, required for ICRP 103 effective dose estimation, are not reported in OLINDA/EXM 1.1 and were assumed to be identical to average total-body dose. Unfortunately, complete urinary data were only available for five human subjects, so the mean TIAC was applied for the remaining four subjects.

The subjects included in the present work all had adrenal pathologies which might affect the estimated absorbed doses. However, we have previously shown that adrenal uptake is blood flow limited both in healthy and pathological adrenals [14], and kinetics are similar in healthy and pathological adrenals with the exception of adrenal carcinoma. Hence, the estimated absorbed dose coefficients can be considered representative for both healthy and non-carcinoma pathological adrenals. Based on our findings, we confirm that [^18^F]CETO is safe for clinical use with respect to radiation dose.

## Conclusions

[^18^F]CETO has a favourable biodistribution in humans for adrenal imaging. The highest absorbed dose coefficients were found for the adrenal glands at approximately 0.1 mGy/MBq. The effective dose for a typical clinical PET examination with 200 MBq [^18^F]CETO is 4 mSv.

## Data Availability

The datasets used and/or analysed during the current study are available from the corresponding author on reasonable request.
